# Intravenous Bolus Fluid Therapy Versus No Fluid Therapy Prior to Pericardiocentesis in Dogs: A Randomized Controlled Trial in 30 Dogs

**DOI:** 10.1111/vec.70073

**Published:** 2026-02-19

**Authors:** Nadine Jones, Karen Humm, Erica W. Tinson

**Affiliations:** ^1^ Veterinary Clinical Science and Services The Royal Veterinary College London UK

**Keywords:** cardiac tamponade, IV fluid bolus, obstructive shock, pericardial effusion, shock index

## Abstract

**Objective:**

To determine the cardiovascular and respiratory effects of bolus IV isotonic crystalloid fluid therapy before pericardiocentesis in dogs with pericardial effusion.

**Design:**

Prospective, randomized, nonblinded clinical trial (January 2021 to November 2022).

**Setting:**

University teaching hospital.

**Animals:**

Thirty dogs diagnosed with pericardial effusion.

**Interventions:**

Dogs were randomized to receive a 10‐mL/kg IV bolus of compound sodium lactate (IV fluid bolus [IVFB] group) or no fluid bolus (no‐IVFB group) over 10 min before pericardiocentesis.

**Measurements and Main Results:**

Cardiovascular parameters, respiratory rate, peripheral blood lactate concentration, and point‐of‐care ultrasound (POCUS) findings were assessed. Shock index (SI) was calculated as heart rate divided by systolic blood pressure. Measurements were recorded at four time points (T): baseline (T0), 15 min later (T1, after fluid bolus for the IVFB group), immediately after pericardiocentesis (T2), and 4 h after pericardiocentesis (T3). Significant decreases in SI were observed in the no‐IVFB group between T1 and T2 and between T1 and T3, and in the IVFB group between T1 and T3 (*p* = 0.034, *p* = 0.003, and *p* = 0.027, respectively). No differences in SI were found between the groups when compared at the same time point. Administration of an IVFB did not result in adverse respiratory effects, as no dogs required supplemental oxygen by T3, and there were no differences in respiratory rate. The no‐IVFB group had a higher incidence of new B‐lines compared with the IVFB group (60% vs. 20%), but the median B‐line score was ≤1 at all POCUS sites at T0 and T3. Twenty‐four dogs survived to hospital discharge with no difference in survival between groups.

**Conclusions:**

Administering an IVFB of 10 mL/kg isotonic crystalloid before pericardiocentesis in dogs with pericardial effusion did not alter SI. This dose appears to be safe, with no adverse respiratory effects seen. However, the current study was underpowered to detect a significant difference, and further studies are needed.

**Abbreviations**
CPA: cardiopulmonary arrest
CSL: compound sodium lactate
CVC:Ao: caudal vena cava‐to‐aorta ratio
HR: heart rate
IVC:Ao: inferior vena cava to aorta ratio
IVFB: intravenous fluid bolus
LVEDP: left ventricular end‐diastolic pressure
No‐IVFB: no intravenous fluid bolus
POCUS: point‐of‐care ultrasound
RR: respiratory rate
SBP: systolic blood pressure
SI: shock index
T: time point

## 1 Introduction

Shock is characterized by a disparity in the supply of oxygen and its demand, leading to insufficient cellular energy production, cell death, and dysfunction of multiple organ systems. The mechanisms that cause this imbalance are classified as hypovolemic, distributive, cardiogenic, obstructive, hypoxic, and metabolic shock [1]. With hypovolemic shock, IV fluid therapy is indicated to restore intravascular volume, whereas in cardiogenic shock, IV fluid therapy is contraindicated.

Pericardial effusion is an uncommon but life‐threatening emergency in dogs that can result in cardiac tamponade and obstructive shock [1]. The cardiovascular effects of the pericardial effusion depend on the speed and volume of fluid accumulation and on the compliance of the pericardial sac [2]. Fluid accumulation results in an increase in intrapericardial pressure, which can exceed the diastolic pressures of the heart chambers; first with the right atrium, then the right ventricle, and then, potentially, the left‐sided heart chambers. This causes collapse of the heart chambers (cardiac tamponade), resulting in a decrease in venous return, decreased preload chamber filling, and, therefore, decreased cardiac output [1]. This obstructive shock may also be compounded by hypovolemia if the pericardial effusion occurs due to hemorrhage.

It may or may not be beneficial to administer an IV fluid bolus (IVFB) to dogs with pericardial effusion, and this has not been evaluated in animals with naturally occurring pericardial effusion. One experimental study in dogs induced pericardial effusion and tamponade in three volume states: hypovolemia due to blood loss, hypervolemia (induced by infusing an IVFB of normal saline and dextran), and euvolemia. The dogs that received an IVFB had improved cardiac output, and the onset of cardiac tamponade was delayed compared with hypovolemic or euvolemic dogs [3]. However, there is also the potential for adverse effects of IVFB therapy in cardiac tamponade. Due to the compression of the heart chambers from pericardial effusion, an increased right heart volume could result in compression of the left side of the heart, with a resultant fall in cardiac output [4].

Although the cardiovascular effects of an IVFB administered to people with pericardial effusion and cardiac tamponade are variable, it has been suggested that an IVFB of 250–500 mL be administered as part of initial stabilization [4, 5]. In another experimental study in dogs, cardiac tamponade resulted in increased left ventricular end‐diastolic pressure (LVEDP), and subsequent volume expansion with an IVFB of synthetic colloid led to a further increase in LVEDP. The study investigators therefore hypothesized that there could be an increased risk for the development of pulmonary edema and dyspnea [6]. Pulmonary edema solely as a consequence of cardiac tamponade has not been described in people [7]. Interestingly, acute‐onset pulmonary edema after pericardial decompression has been reported in people, though the pathophysiological mechanism remains unclear [8]. The incidence of dyspnea or pulmonary edema after pericardiocentesis has not been reported in dogs, and it is unknown whether pericardiocentesis or IVFB therapy in these patients could result in dyspnea or pulmonary edema.

The current study aimed to determine whether an IVFB administered to dogs with pericardial effusion before pericardiocentesis would result in improved perfusion parameters, lactate concentration, and shock index (SI) compared with dogs that did not receive an IVFB before pericardiocentesis. We hypothesized that there would be no difference in heart rate (HR), systolic blood pressure (SBP), lactate, or SI between the two groups. Secondary aims were to evaluate whether dogs developed pulmonary edema or increased respiratory rate (RR) either as a consequence of pericardiocentesis or as a result of an IVFB. We hypothesized that there would be no difference in RR as a result of the IVFB or pericardiocentesis.

## 2 Materials and Methods

This was a prospective, randomized clinical trial carried out from January 2021 to November 2022. Dogs presenting to a university teaching hospital with pericardial effusion requiring pericardiocentesis were considered for inclusion, and informed owner consent was obtained. Exclusion criteria were a lack of clinical indication for pericardiocentesis, pericardial effusion due to left atrial rupture or coagulopathy, lack of owner consent for treatment or study enrollment, and exclusion by the clinician based on the need for immediate pericardiocentesis, which precluded owner consent from being obtained.

The design of the current study was approved by the Clinical Research and Ethical Review Board (reference number 2020 1964‐3) at the Queen Mother Hospital for Animals at the Royal Veterinary College. An Animal Test Certificate from the Veterinary Medicine Directorate was issued for the use of compound sodium lactate (CSL; ATC‐S‐146)^1^ as an IVFB. A sample size calculation could not be performed because the effect of IVFB in dogs with pericardial effusion has not been evaluated clinically. Based on historical case numbers, a recruitment of 30 dogs was deemed achievable in a 2‐year time frame. Once enrolled, dogs were randomized to either receive an IVFB (IVFB group) or not to receive an IVFB (no‐IVFB group), with 15 dogs per treatment group. Before the study began, patients were randomized into 1:1 blocks with a random number generator, and group allocation and data collection sheets were then stored in sealed, numbered envelopes.

**FIGURE 1:**
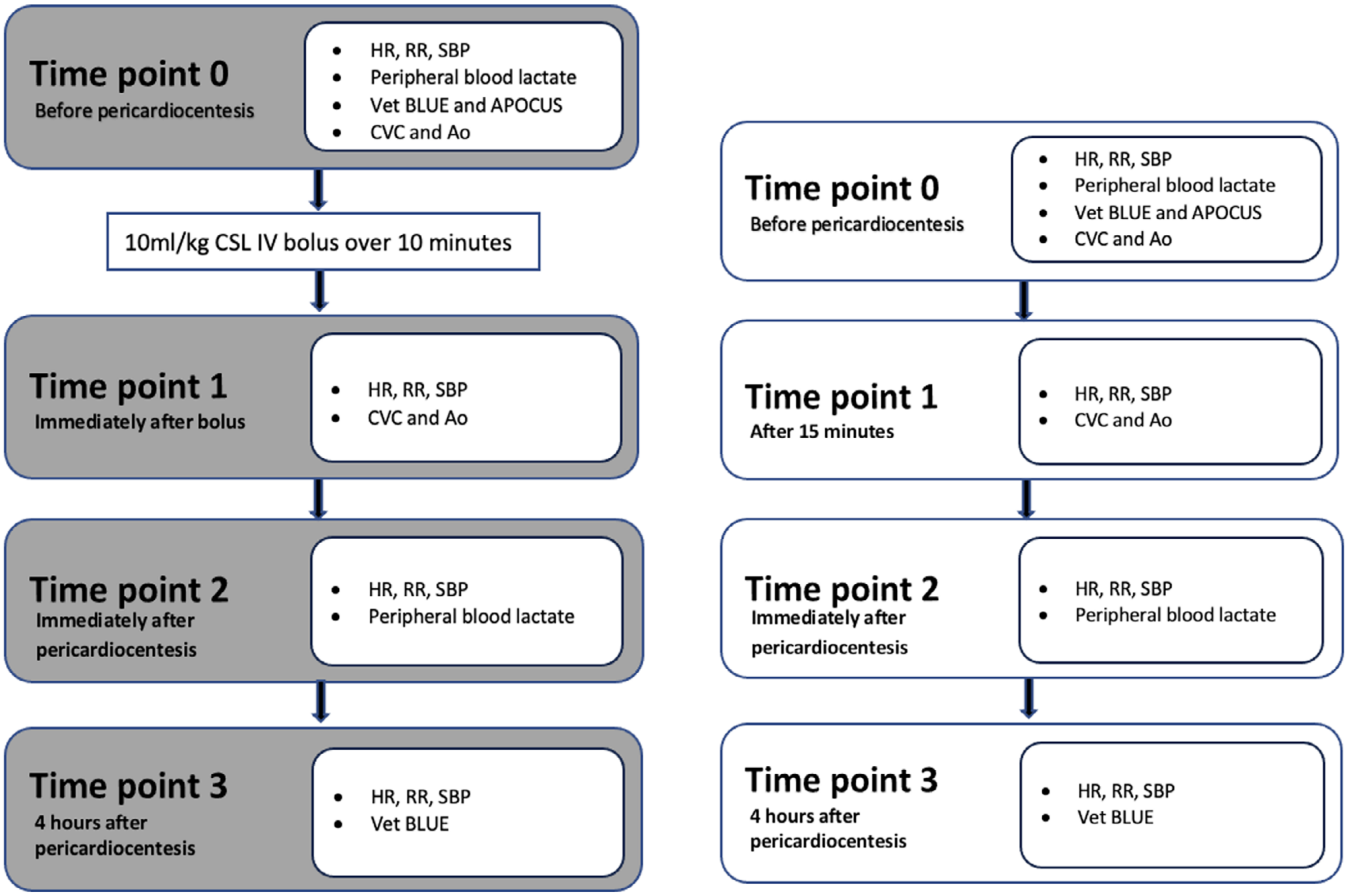
Flow chart of events after study enrollment in 30 dogs with pericardial effusion requiring pericardiocentesis that were randomized to receive an IVFB (left) versus no IVFB (right). Ao, aorta; APOCUS, abdominal point‐of‐care ultrasound; CSL, compound sodium lactate; CVC, caudal vena cava; HR, heart rate; IVFB, IV fluid bolus; RR, respiratory rate; SBP, systolic blood pressure; Vet BLUE, Veterinary Bedside Lung Ultrasound Examination.

Once dogs were enrolled, the next sealed, numbered envelope was selected. Figure 1 shows a flow chart of the clinical trial for each treatment group. At the first time point (time point 0 [T0]), all the dogs underwent physical examination. The HR and RR were recorded. A noninvasive SBP measurement was obtained via Doppler and sphygmomanometer. Measurements were obtained using the same method as outlined in the ACVIM consensus statement [9]. All patients had an IV cannula placed, and a venous blood sample was obtained for lactate measurement^2^.

Point‐of‐care ultrasound (POCUS) of the thorax and abdomen was performed as previously described [10, 11]. Data recorded included abdominal free‐fluid score and the presence or absence of gallbladder wall edema, cardiac tamponade, and pleural effusion. For the lungs, a previously described thoracic POCUS protocol^3^ was used to evaluate for ultrasound artifacts (B‐lines) [11]. The patient was positioned either in sternal recumbency or standing according to their preference. The same ultrasound machine^4^ was used to scan all animals, and a curvilinear 8‐mHz ultrasound probe was used in B mode. For each lung site, the image obtained was graded as 0 (no B‐lines), 1 (one single B‐line), 2 (two single B‐lines), 3 (three single B‐lines), 4 (more than three B‐lines noted as individual B‐lines), or confluent B‐lines (recorded as five), with eight sites evaluated in total, four per hemithorax. New B‐lines were defined as any number of B‐lines at any site compared with an initial score of 0 for that site at T0. POCUS was performed by emergency and critical care residents with previous training and experience who were not blinded to the treatment groups. Caudal vena cava and aorta measurements were performed using ultrasound by a single investigator (NJ) who was not blinded to the patient treatment groups. The caudal vena cava and aorta were visualized via the right paralumbar view as previously described, and the internal diameter of the vessels was measured to obtain the caudal vena cava to aorta (CVC:Ao) ratio [12].

If allocated to the IVFB group, dogs received a 10‐mL/kg IVFB of CSL over 10 min. This was delivered by a fluid pump or, if the dog weighed >17 kg, via a pressure bag. Fluid would be removed from the bag of CSL to ensure the correct volume was delivered (e.g., 20 mL removed from a 250‐mL fluid bag to deliver 230 mL for a 23‐kg dog). If the pressure bag was used, we monitored the flow of the isotonic crystalloid, adjusting as necessary, to ensure the fluid was administered within the timeframe. Fifteen minutes after the initial assessment (time point 1 [T1]), RR, HR, and SBP measurements were performed. All dogs then underwent pericardiocentesis and had HR, RR, and SBP recorded, along with a repeat lactate measurement (time point 2 [T2]). The pericardiocentesis technique (needle pericardiocentesis or pericardial catheter placement) was chosen by the attending clinician. Any sedatives and doses used were also chosen and recorded by the clinician. Four hours after pericardiocentesis, HR, RR, and SBP were recorded, and a repeat thoracic POCUS was performed (time point 3 [T3]). If a dog was noted to be panting and an accurate RR could not be recorded, that dog's RR was not included for further analysis. If oxygen supplementation was required at T3 due to dyspnea, this was recorded. SI was defined as HR (/min)/SBP (mm Hg) and was calculated at each time point for each dog. An SI *>*0.9 was considered abnormal [13].

Other recorded data included any medications administered during the study period (e.g., antiarrhythmic drugs), volume of pericardial effusion drained, whether drainage was achieved by centesis only (e.g., butterfly catheter or IV cannula) or placement of a pericardial catheter, any complications noted during the procedure, and whether additional IVFBs were administered to dogs in either group. The attending clinician could decide to administer an IVFB, and the reason for administration was recorded. The cause of the pericardial effusion, as determined by echocardiography or computed tomography (etiology then classified as either neoplasia or presumed idiopathic), performed by a board‐certified cardiologist or diagnostic imager, was recorded. Outcomes were recorded as survival to discharge, death by cardiopulmonary arrest (CPA), or euthanasia.

### 2.1 Statistical Analysis

Continuous data were assessed for normality using the Shapiro–Wilk test. Normal data were described using the mean (SD), and nonnormal data were described using the median (range). Normally distributed data were compared between groups using *t*‐tests and Mann–Whitney *U*‐tests if data were not normally distributed. Mann–Whitney *U*‐tests were used for intergroup comparisons (e.g., to compare the IVFB and no‐IVFB groups with each other at the same time point). For paired comparisons over time (e.g., comparison of the same group at different time points), the Wilcoxon signed‐rank test was used. A *χ* ^2^ test was used to compare categorical data (survival vs. nonsurvival). Intention‐to‐treat analysis was performed, whereby if a dog was randomized to the no‐IVFB group but then received a fluid bolus, the dog remained in the no‐IVFB group for analysis. A *p*‐value of <0.05 was considered statistically significant. A post hoc analysis was performed to determine the effect size for SI and lactate concentration, and a power calculation was performed to determine the power of the study based on sample size. Statistical analysis was performed using commercially available software^5^.

## 3 Results

### 3.1 Population Baseline Characteristics

Seventy dogs with pericardial effusion were presented to the Queen Mother Hospital for Animals of the Royal Veterinary College between January 2021 and November 2022. Forty dogs were excluded due to reasons including pericardiocentesis being deemed unnecessary (either due to the pericardial effusion being too small in volume to drain or pericardiocentesis performed at the primary care veterinarian's facility before referral [*n* = 15]), euthanasia performed rather than pursuing pericardiocentesis and treatment (*n* = 10), clinician exclusion due to the need for rapid pericardiocentesis, which precluded discussion with the owner regarding the study (*n* = 5), consent for study enrollment declined by the owner (*n* = 4), pericardial effusion due to coagulopathy (*n* = 2) or left atrial rupture (*n* = 1), study investigator not available to assist (*n* = 1), patient deceased on arrival (*n* = 1), and attending clinician not aware of the study (*n* = 1).

Thirty dogs were enrolled in the study, and 15 dogs were randomized to each group. Breeds included French Bulldog (*n* = 5), Labrador Retriever (*n* = 4), Golden Retriever (*n* = 3), Cocker Spaniel (*n* = 3), Staffordshire Bull Terrier (*n* = 3), Border Collie (*n* = 2), Greyhound (*n* = 2), crossbreed (*n* = 2), American Bulldog (*n* = 1), British Bulldog (*n* = 1), Rottweiler (*n* = 1), German Shepherd Dog (*n* = 1), Dalmatian (*n* = 1), and Shih‐Tzu (*n* = 1). Twenty‐two dogs were male, 18 of which were neutered. Eight dogs were female, seven of which were neutered. The mean age of all dogs was 122 months (±SD 29.4). There was no difference in mean age between the two groups (123 and 118 months for the no‐IVFB and IVFB groups, respectively) (*p* = 0.62). There was no difference in median body weight between the two groups (24.5 kg [6.8–37.8 kg] and 26.3 kg [13.4–49.3 kg] for the no‐IVFB versus IVFB groups, respectively; *p* = 0.82).

At T0, POCUS of the abdomen was performed in 30 dogs and of the thorax in 28 dogs. Two dogs did not undergo POCUS of the thorax due to the need for immediate pericardiocentesis after tamponade was identified in one case and because of aggressive patient behavior in the other case. The median abdominal fluid score was 2 (range: 0–4) out of a possible 4. Twenty‐seven dogs had cardiac tamponade. Of the three that did not have cardiac tamponade, two were in the no‐IVFB group and one was in the IVFB group. Ten dogs had the presence of gallbladder edema on abdominal POCUS—four in the no‐IVFB group and six in the IVFB group.

One patient was anesthetized with propofol, intubated, and maintained on a propofol constant rate infusion for total IV anesthesia for pericardiocentesis. The remaining 29 dogs underwent sedation for the procedure. The most frequently administered drug was butorphanol (*n* = 19/29), followed by propofol (*n* = 16/29), midazolam (*n* = 11/29), alfaxalone (*n* = 9/29), methadone (*n* = 8/29), and medetomidine (*n* = 3/29). Clinicians elected to place a pericardial drain in 22 dogs and to perform pericardiocentesis with a butterfly catheter or IV cannula in eight dogs. The volume of pericardial fluid drained ranged from 0.02 to 39.44 mL/kg (median: 6.66 and 9.66 mL/kg for the no‐IVFB and IVFB groups, respectively).

Complications encountered during pericardiocentesis included placement of a pericardial catheter into the heart and draining of blood (*n* = 2, one in each treatment group), hypotension managed with the administration of an IVFB (*n* = 2, no‐IVFB group), ventricular tachycardia after pericardiocentesis necessitating a lidocaine constant rate infusion (*n* = 2, both in the IVFB group), and CPA after T2 (*n* = 1, no‐IVFB group). The two dogs with blood drained from the heart had 172 and 5 mL removed. The dog with 172 mL drained (equivalent to 16 mL/kg) received 23 mL/kg CSL as an IVFB but had initially been randomized to the no‐IVFB group. The pericardial catheter was removed and replaced in the correct position for pericardiocentesis, and 50 mL of pericardial effusion was removed. The dog that had 5 mL of blood drained (equivalent to 0.1 mL/kg) did not receive any additional IV fluids; the pericardial catheter was removed, and needle pericardiocentesis was performed to drain the effusion. The second dog in the no‐IVFB group that received an IVFB was tachycardic and hypotensive at T0 and T1 (SBP of 65 and 55 mm Hg, respectively) and therefore received a total of 30 mL/kg of CSL. This dog also had a recent history of diarrhea and continued to pass hemorrhagic diarrhea in the hospital after pericardiocentesis. The dog that suffered the CPA had undergone pericardiocentesis without any apparent issue, and resuscitation was not performed, per the owner's wishes. The cause of the CPA was unknown. None of the other dogs received additional fluids between the other time points.

Subsequent investigations in 15 cases revealed a cardiac mass identified on either specialist echocardiography or computed tomography. Diagnostic imaging was suggestive of neoplasia in an additional four cases: suspected mesothelioma (*n* = 3) and based on evidence of lung metastasis (*n* = 1). Therefore, a total of 19 cases (63%) were diagnosed with neoplastic etiology for the pericardial effusion, and the remaining 11 cases (37%) were presumed to be of idiopathic etiology.

### 3.2 Cardiovascular Effects

**FIGURE 2:**
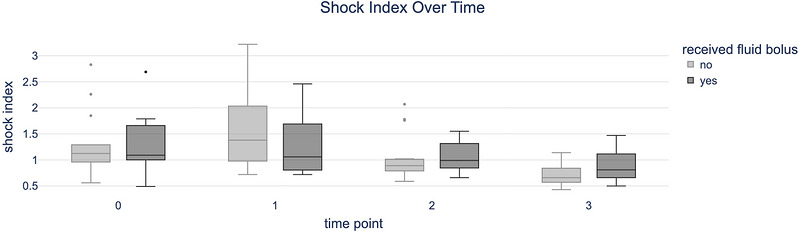
Box‐and‐whisker plots showing change in shock index between time points 0 and 3 in 30 dogs with pericardial effusion requiring pericardiocentesis that were randomized to receive an IV fluid bolus or not to receive an IV fluid bolus. The box represents data from the first to third quartile, while the median is represented by the horizontal line within each box. The whiskers represent the range, up to 1.5 times the interquartile range. Dots represent individual outliers.

Table 1 shows the HR, SBP, and SI across the time points, and SI trends are shown in Figure 2. SI could be calculated in 28 dogs at T0. Of these, 23 had an SI >0.9 (82% [no‐IVFB, *n* = 11; IVFB, *n* = 12]). When groups were compared at the same time point, the only difference was in HR at T3, which was significantly higher in the IVFB group (*p* = 0.047). Over time, significant changes were observed in the no‐IVFB group, with an increase in SBP between T1 and T2 (*p* = 0.04) and between T1 and T3 (*p* = 0.008), a decrease in HR between T1 and T2 (*p* = 0.004) and between T1 and T3 (*p* = 0.003), and a decrease in SI between T1 and T2 (*p* = 0.034) and between T1 and T3 (*p* = 0.003). The only significant difference found for the IVFB group was a decrease in SI between T1 and T3 (*p* = 0.027).

**TABLE 1:** Perfusion parameters, RR, lactate concentration, and CVC:Ao ratio in 30 dogs with pericardial effusion requiring pericardiocentesis that were randomized to receive an IVFB versus no IVFB.

Parameters	T0 (no‐IVFB)	T0 (IVFB)	T1 (no‐IVFB)	T1 (IVFB)	T2 (no‐IVFB)	T2 (IVFB)	T3 (no‐IVFB)	T3 (IVFB)
HR (/min)	138 (±30)	152 (±31)	146 (±27)^b^	135 (±28)	121 (±26)^b^	123 (±26)	84 (64–188)^a^, ^b^	121 (±35)^a^
SBP (mm Hg)	121 (±33)	130 (±32)	103 (±31)^b^	116 (±29)	127 (±27)^b^	121 (±14)	137 (±23)^b^	136 (±23)
Number of hypotensive dogs (SBP <100 mm Hg)	4/14	3/14	7/12	4/11	3/15	1/12	0/10	0/12
SI	1.12 (0.56–2.83)	1.29 (±0.54)	1.56 (±0.74)^b^	1.28 (±0.55)^c^	0.89 (0.59–2.07)^b^	1.06 (±0.28)	0.73 (±0.23)^b^	0.9 (±0.33)^c^
SI >0.9	11/14	12/14	9/11	7/11	7/15	9/12	2/10	3/13
RR (/min)	30 (±6)	36 (±8)	26 (18–48)	30 (±13)	26 (16–65)	28 (20–40)	24 (16–60)	29 (16–60)
Lactate (mmol/L)	2.2 (1.3–8.8)	2.6 (1.3–9.5)	n/a	n/a	2.1 (0.5–8.4)	1.9 (0.8–3.6)	n/a	n/a
CVC:Ao ratio	1.19 (±0.26)	1.1 (±0.22)	1.0 (±0.23)	1.02 (±0.22)	n/a	n/a	n/a	n/a

*Note*: Data reported as median (range) if not normally distributed or mean (SD) if normally distributed. SI calculated as HR/SBP.

Abbreviations: CVC:Ao, caudal vena cava‐to‐aorta ratio; HR, heart rate; IVFB, IV fluid bolus; RR, respiratory rate; SBP, systolic blood pressure; SI, shock index; T0, time point 0; T1, time point 1; T2, time point 2; T3, time point 3.

^a^ Significant difference between the no‐IVFB and IVFB groups at T3.

^b^ Significant difference between two time points (between T1 and T2 and between T1 and T3) for the no‐IVFB group in SBP, HR, and SI.

^c^ Significant difference between two time points (between T1 and T3) for the IVFB group.

**FIGURE 3:**
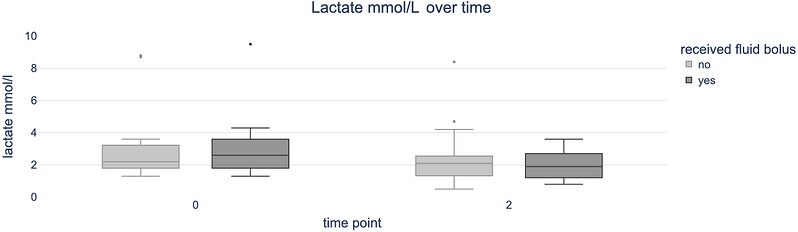
Box‐and‐whisker plots showing lactate between time points 0 and 2 in 30 dogs with pericardial effusion requiring pericardiocentesis that were randomized to receive an IV fluid bolus or not to receive an IV fluid bolus. The box represents data from the first to third quartile, while the median is represented by the horizontal line within each box. The whiskers represent the range, up to 1.5 times the interquartile range. Dots represent individual outliers.

In the IVFB group, the median lactate concentration decreased from 2.6 to 1.9 mmol/L between T0 and T2, and in the no‐IVFB group, the median lactate concentration decreased from 2.2 to 2.1 mmol/L (Figure 3). There were no differences in lactate concentrations between the two time points, either for the IVFB group (*p* = 0.07) or for the no‐IVFB group (*p* = 0.054).

### 3.3 RR and Thoracic POCUS

Nine dogs were panting at T0, and therefore an accurate RR was not recorded; these cases were excluded from further analysis of RR. RR was not significantly different between the groups at any time point (Table 1).

At T0, POCUS of the thorax was performed in 28 dogs; it could not be performed in two dogs due to aggressive behavior (*n* = 1) and due to the need for rapid pericardiocentesis with patient decompensation (*n* = 1). Eleven dogs had a pleural effusion (five in the no‐IVFB group and six in the IVFB group), and B‐lines were present in five dogs (two in the no‐IVFB group and three in the IVFB group). At T3, 23 dogs had repeat POCUS of the thorax. Seven patients did not have repeat thoracic POCUS at T3 due to euthanasia or cardiac arrest (*n* = 4), unknown reason (*n* = 2), or aggressive behavior (*n* = 1). The median B‐line score per site at T0 was 0 for all sites, and for T3, the median B‐line score was 0 for all sites, except for the left middle lung lobe region and left perihilar lung lobe region (median B‐line score of 1 for both). In the no‐IVFB group, new B‐lines (defined as any number of B‐lines at any site compared with an initial score of 0 at T0) were recorded in nine dogs (60%) (middle: *n* = 3, perihilar: *n* = 3, cranial: *n* = 2, and caudodorsal: *n* = 1). In the IVFB group, new B‐lines were recorded in three dogs (20%) (middle: *n* = 1, perihilar: *n* = 1, cranial: *n* = 1). None of the patients required supplemental oxygen therapy at T3.

### 3.4 CVC:Ao Ratio

CVC:Ao ratio was measured in 22 dogs at T0 (11 in each group) and T1 (11 in each group) (Table 1). There was no difference between groups in the ratio at T0 (*p* = 0.40) or T1 (*p* = 0.829). There was no difference for either the no‐IVFB or the IVFB group between T0 and T1 (*p* = 0.08 and *p* = 0.4, respectively).

### 3.5 Outcome

Twenty‐four dogs survived to hospital discharge: 11 from the no‐IVFB group and 13 from the IVFB group. Of the six nonsurvivors, five were euthanized and one suffered a CPA. There was no difference in survival between the two groups (*p* = 0.68).

### 3.6 Post Hoc Analysis

The effect size for SI at T1 was 0.43, which represents a standardized difference between the two means and indicates a small to medium effect size. The power for SI at T1 was 16%. To detect a clinically significant difference between the two groups at T1 with sufficient power of 80%, approximately 86 dogs per group would be needed. For lactate at T2, the effect size was 0.35, also indicating a small to medium effect size, with a power of 15%. To achieve 80% power to detect a clinically significant difference in lactate between the two groups at T2, approximately 64 dogs per group would be needed.

### 3.7 Intention‐to‐Treat Analysis Versus Exclusion of No‐IVFB Dogs Given IVFB

Two dogs in the no‐IVFB group received ≥1 IVFB as decided and directed by the attending clinician. When these dogs were excluded from analysis, differences in HR between T2 and T3 in the no‐IVFB group became significant (*p* = 0.019), with a lower HR seen at T3, whereas no significance was previously observed (*p* = 0.123). Additionally, the previously significant decrease in SI between T1 and T2 in the no‐IVFB group was no longer significant (*p* = 0.07). There were still no differences in lactate or CVC:Ao ratio between the groups or across time points.

## 4 Discussion

This prospective study examined the effect of an IVFB in dogs with pericardial effusion before undergoing pericardiocentesis. We did not find a discernible advantage in administering an IVFB, as evidenced by a lack of a significant change in SI between T0 and T1, supporting our initial hypothesis. However, our study was underpowered to detect a difference in SI at T1. We did find that pericardiocentesis, the mainstay of treatment for cardiac tamponade, resulted in a decrease in SI for both groups.

The current study is the first to describe SI in dogs with pericardial effusion, finding that it was increased in 23 of 28 (82%) dogs on presentation. Dogs in the no‐IVFB group had an increase in SI from T0 to T1, which may suggest worsening or ongoing shock; however, this difference was not significant. There were no significant differences in SI between the groups when compared with each other at the same time point, and no significant differences were found for either group between T0 and T1, suggesting that the IVFB did not influence the SI or that the study was underpowered to detect an impact. However, there were differences in SI when comparing the no‐IVFB group from T1 to T2 and from T1 to T3 (median values of 1.56 and 0.89, respectively; mean value of 0.73 for T1, T2, and T3). For the IVFB group, the only significant difference in SI was found between T1 and T3 (mean value decreased from 1.28 to 0.9). By T3, the mean SI was ≤0.9 in both treatment groups. These results show that there was a resolution of shock for both treatment groups due to the effect of pericardiocentesis.

SI was used in this study because HR and SBP are perfusion parameters that can be readily measured, with SI easily calculated from these values. SI has been evaluated as an indicator of cardiovascular compromise in both people and dogs and may be used to identify occult shock [13, 14, 15, 16]. It has been shown to be inversely related to cardiac index and mean arterial pressure in experimental pig studies when inducing hypovolemia via blood loss [17]. A cutoff value of >0.9 has been proposed as a triage tool in dogs, with relatively high sensitivity but low specificity for shock (92% and 50%, respectively) [13]. SI has thus far been used to evaluate dogs with hypovolemic shock after hemorrhage, vehicular trauma, or after blood donation, but it has not been evaluated in dogs with pericardial effusion [13, 18, 19]. Our study showed a downward trend in SI toward normal after stabilization with pericardiocentesis, regardless of IVFB administration. Limitations of using SI include potential variations in normal baseline HR according to breed and the use of sedative drugs to facilitate pericardiocentesis, which may have affected both HR and SBP when measured at T2 but which, by T3, would have been unlikely to still be affecting the dogs [20].

Lactate is a metabolite that is produced both under healthy resting conditions and in states of shock [21]. In shock, inadequate oxygen delivery to tissues leads to impaired mitochondrial function and increased anaerobic metabolism [21]. Alternative mechanisms for hyperlactatemia in shock states include acceleration of aerobic glycolysis due to inflammation and release of endogenous catecholamines stimulating glycolysis and glycogenolysis [22]. Plasma lactate and its change in concentration over time are potential predictors of mortality in both people and dogs in shock [23, 24, 25]. A lactate concentration of <2.5 mmol/L has been established as normal for healthy dogs, and various studies have defined a lactate >2.0 or 2.5 mmol/L as abnormal and consistent with shock [14, 24, 26]. In the current study, the starting median lactate values were 2.2 and 2.6 mmol/L for the no‐IVFB and IVFB groups, respectively, and therefore similar to the upper end of the reference range for what has been previously described in healthy dogs [26]. There were no significant changes in lactate concentration for either group between T0 and T2, although it is interesting to note that the median lactate decreased from 2.6 to 1.9 mmol/L in the IVFB group, which could reflect improved perfusion due to the IVFB. The absence of a significant difference may be attributed to the small sample size in each group, as evidenced by our low post hoc power calculation of 15%, which suggests a relatively low likelihood of detecting a true effect even when one exists, thereby increasing the risk of type II error. It is also possible that 10 mL/kg was an insufficient volume of IVFB to improve tissue perfusion, or there could have been a lag in improved perfusion and lactate clearance.

Dogs in the current study may also have been hyperlactatemic for other reasons, such as a result of neoplasia (i.e., type B hyperlactatemia), although at T2, the median value for lactate concentration was within the normal interval for both groups. Also, CSL, which contains lactate, was used as the IVFB. Therefore, in theory, an IVFB of CSL could increase plasma lactate concentration. It has been shown that blood lactate increases with very large boluses of 180 mL/kg in healthy dogs, although smaller boluses of 10 mL/kg were not evaluated [27].

People with pericardial effusion have a variable response to the administration of IV fluids [4]. An increase in cardiac index was observed in 47% of human patients with pericardial effusion after the administration of 500 mL of normal saline over 10 min; however, 31% had a decrease in cardiac index, and 22% had no change [4]. The authors identified that patients with an SBP of <100 mm Hg before a fluid bolus were more likely to benefit from an IVFB, as determined by an increase in their cardiac index [4]. A smaller study found that a lower SBP on presentation was a significant predictor for an increase in cardiac index in people administered an IVFB of 500 mL of normal saline [28]. A recent review of the emergency treatment of pericardial effusion resulting in tamponade suggests the administration of 250–500 mL of IV fluids over 10 min in patients with an SBP of <100 mm Hg [5]. Administering an IV synthetic colloid to dogs before inducing a pericardial effusion in an experimental setting resulted in improved cardiac output compared with euvolemia or hypovolemia [29]. Measurement of cardiac output is often not feasible outside of experimental studies; therefore, surrogate markers of perfusion, such as HR, SBP, and SI, such as those in our study, are used. There were similar numbers of hypotensive dogs in each group at T0 in the current study, but more dogs were hypotensive at T1 in the no‐IVFB group compared with the IVFB group (58% vs. 36%). After pericardiocentesis at T2, there were still more hypotensive dogs in the no‐IVFB group compared with the IVFB group (20% vs. 8%). It may be that there is a benefit in the administration of an IVFB in dogs with pericardial effusion that are hypotensive on presentation; however, the number of dogs in our study was too small to perform further analysis on this subgroup. Our current results do not suggest that an IVFB is beneficial in stabilizing dogs with pericardial effusion; however, the study was underpowered to detect a difference between treatment groups. A post hoc power for SI at the main time point of interest (T1) revealed a power of 16% for detecting a difference between the two groups, indicating a relatively low probability of detecting a true effect, if one existed. Although we cannot conclude that an IVFB is beneficial, its administration is rapid and can be done while preparing the dog for pericardiocentesis. It is also important to note that by T3, none of the dogs were hypotensive, indicating that pericardiocentesis will effectively treat the shock state resulting from pericardial effusion, whether a fluid bolus is administered or not.

No increase in RR was observed between the two groups at any time point, and none of the dogs required oxygen supplementation at T3, indicating no adverse effects on the respiratory system as a result of pericardiocentesis or IVFB administration in this population. Lung ultrasound is highly sensitive and specific in the assessment of pulmonary edema in people and in the diagnosis of cardiogenic pulmonary edema in dogs [30, 31]. The median B‐line score for all lung sites evaluated by thoracic POCUS at T0 was 0 for both groups, with two sites subsequently having an increase in median B‐line score from 0 to 1 at T3 (both sites for the no‐IVFB group). However, this increase is not considered clinically relevant as single B‐lines have been reported in dogs without respiratory disease and with radiographically normal lungs [11]. Of note, more dogs in the no‐IVFB group (9/15 [60%]) developed new B‐lines compared with the IVFB group (3/15 [20%]). The cause of this higher incidence of newly developed B‐lines in the no‐IVFB group is unclear but, again, is not considered to be clinically relevant. In people, a condition termed “pericardial decompression syndrome” describes paradoxical hemodynamic deterioration or development of pulmonary edema after pericardiocentesis. It is rare and is associated with high mortality rates of 22.5% [8]. There are several theories as to how pericardiocentesis could result in this acute deterioration, including rapid right ventricular expansion or myocardial stunning [7]. Pericardial decompression syndrome has not been reported in dogs. One dog in the current study died suddenly after pericardiocentesis. The cause of death was unknown, and a postmortem examination was not performed. It is interesting to note that the prevalence of gallbladder edema in dogs with pericardial effusion was 33%. A previous study describing gallbladder changes in dogs with cardiac disease identified pericardial effusion as the most common cause of gallbladder wall edema [32].

In people, the inferior vena cava to aorta (IVC:Ao) ratio has been found to correlate with intravascular volume status [33]. The canine equivalent, the CVC:Ao ratio, has gained attention for its potential role in assessing fluid responsiveness and hemodynamic status in healthy dogs, in dogs administered furosemide to induce hypovolemia, and in dogs hospitalized in an ICU [12, 34, 35]. A lower ratio is associated with hypovolemia, and a higher ratio is potentially indicative of hypervolemia [34, 36]. The current study found that the CVC:Ao ratio at T0 was increased (mean values of 1.19 and 1.1 for the no‐IVFB and IVFB groups, respectively) compared with the reported mean CVC:Ao ratio of 0.96 that was obtained in healthy dogs at the same paralumbar site [12].

The increased ratio in our study resulted from the decreased venous return to the right atrium, caused by the compression from the pericardial effusion. This led to caudal vena cava distension and, consequently, an increased CVC:Ao ratio. In both groups, the mean CVC:Ao ratio decreased at T1 (to 1.0 and 1.02 for the no‐IVFB and IVFB groups, respectively). The reason for this decrease in the ratio is unclear but may be expected with relief of the pericardial effusion, allowing increased venous return with improved right heart filling and therefore decreased dilation of the caudal vena cava. However, T1 measurements were performed before decompression, when the CVC:Ao ratio might be expected to increase in the IVFB group, given the increased preload. It is possible that a 10‐mL/kg bolus is an insufficient volume to create a measurable difference. This theory is supported by a study in people that failed to find a significant change in the IVC:Ao ratio when a 500‐mL IVFB was administered after blood donation [37]. Furthermore, it has been suggested that assessing volume status with ultrasound is not predictive of a patient's response to fluid loading [38]. The inferior vena cava and caudal vena cava are compliant vessels that can experience changes in their diameter according to the respiratory cycle [35, 39]. However, the caudal vena cava diameter is less likely to change with the respiratory cycle in patients with pericardial effusion due to the persistently increased right atrial pressure [38]. Therefore, the respiratory cycle effects on the measurement of the CVC:Ao ratio are less likely to be a factor in the current study.

Important limitations of the current study include the lack of blinding of the person measuring the CVC:Ao, as well as the lack of review of the measurements by a board‐certified diagnostic imager. Future studies could include assessing the CVC:Ao ratio after pericardiocentesis. Our study has several other limitations. Despite its prospective nature, some values were missing from our dataset. Thirty dogs were recruited, and this may have been underpowered to detect a difference between the two treatment groups. Five patients were not enrolled into the study on presentation due to the perceived need for immediate pericardiocentesis; therefore, we may have missed a subset of dogs with hypotension or impending cardiac arrest that may have benefited from intravascular volume expansion. Two dogs in the no‐IVFB group ultimately received IV fluids as decided by the attending clinician. One dog had the pericardial catheter placed in the heart, and 16 mL/kg of blood was removed, resulting in hypotension. The second dog was passing diarrhea and was hypotensive at T0 and T1 and therefore also received fluid boluses of CSL totaling 30 mL/kg. These dogs were analyzed as part of the no‐IVFB group, as we planned an intention‐to‐treat analysis. We then removed these two dogs from analysis and compared the remaining no‐IVFB group to the IVFB group. The significant difference in SI between T1 and T2 was lost for the no‐IVFB group. Future studies with more dogs enrolled should be considered to further investigate the cardiovascular effects of an IVFB in dogs.

Perfusion parameters assessed in the current study included SBP and SI, but it is possible to have microcirculatory dysfunction and shock with normal hemodynamic parameters. BP measurement was performed via a noninvasive Doppler technique, which is not as accurate as the gold standard invasive BP monitoring. However, noninvasive measurements are more practical in an emergency setting, such as during the rapid assessment of a dog with pericardial effusion. Lactate as a marker of perfusion in shock states has limitations, given that it can be normal in patients in shock or increased due to other factors, such as neoplasia [13, 21]. Thoracic POCUS was used to evaluate the number of B‐lines to indicate pulmonary edema. A previous study found that the identification of B‐lines in dogs presenting with dyspnea had a good sensitivity of 83% for the diagnosis of cardiogenic pulmonary edema when compared with thoracic radiographs, but a poorer specificity of 70% [31]. It is possible that dogs in our study developed new B‐lines due to other reasons, such as aspiration and pneumonitis. Furthermore, the previous study identified five dogs with a final diagnosis of cardiogenic pulmonary edema in which no B‐lines were identified on lung ultrasound [31]. Therefore, false negatives are possible with the use of thoracic POCUS.

In conclusion, the administration of a 10‐mL/kg IVFB of an isotonic crystalloid before pericardiocentesis did not significantly improve the SI in dogs with pericardial effusion. However, the current study was underpowered to detect these differences, if they exist. Recognizing this, an IVFB did not result in any adverse respiratory effects and can be considered a safe option during stabilization before pericardiocentesis, particularly in hypotensive patients.

## Author Contributions

**Nadine Jones**: conceptualization, data curation, formal analysis, investigation, methodology, writing – original draft. **Karen Humm**: conceptualization, investigation, methodology, writing – review and editing. **Erica W. Tinson**: conceptualization, investigation, writing – review and editing.

## Conflicts of Interest

The authors declare no conflicts of interest.

## Endnotes

1 Aquapharm 11 Hartmann's solution for infusion, Animalcare Ltd, York, UK.

2 Radiometer ABL90 series.

3 Veterinary Bedside Lung Ultrasound Examination, Vet BLUE.

4 Sonosite VetMed, Sonosite Inc, Bothell, WA.

5 Python 3.7.13 (2022).
